# Fibromyalgia Rapid Screening Tool (FiRST): Arabic Translation and Cross-Cultural Adaptation and Validation

**DOI:** 10.3390/healthcare11070961

**Published:** 2023-03-28

**Authors:** Shiekha S. AlAujan, Haya M. Almalag, Ghadah A. Assiri, Faris A. Alodaibi, Mohammed A. Omair

**Affiliations:** 1 Clinical Pharmacy Department, College of Pharmacy, King Saud University, Riyadh 11362, Saudi Arabia; 2 Rehabilitation Sciences Department, College of Applied Medical Sciences, King Saud University, Riyadh 11362, Saudi Arabia; 3 Rheumatology Unit, Department of Medicine, King Saud University, Riyadh 11362, Saudi Arabia

**Keywords:** translation, cross-cultural adaptation, validation, fibromyalgia, Arabic

## Abstract

**Background:** Fibromyalgia (FM), a complex neurological disorder, has multiple consequences for the patient. To diagnose patients, healthcare practitioners use multiple diagnostic questionnaires. However, Arabic translated or validated tools are lacking. This study aimed to translate and validate the Fibromyalgia Rapid Screening Tool (FiRST) into the Arabic language. **Methods:** Forward and backward translations of the FiRST were conducted by two Arabic translators and two English-certified translators. The survey was piloted (n = 5) and subjected to cognitive interviews and psychometric analysis. Patients were recruited from a university hospital in Riyadh and an FM support group in Saudi Arabia. The internal consistency, factor analysis, and test–retest correlations were evaluated. **Results:** This study included 46 patients. The stepwise translation process resulted in minor edits related to the use of synonyms to the survey items. The translated survey had a good internal consistency and test–retest correlation, with a Cronbach’s alpha of 0.7 and Pearson’s correlation coefficient of 0.79 (*p*-value < 0.001), respectively. The survey was factorable into two themes: generalized symptoms and more specific sensations. **Conclusions:** The Arabic FiRST is a simple, valid, and reliable tool to diagnose patients with FM in different settings.

## 1. Introduction

According to the American College of Rheumatology (ACR), fibromyalgia (FM) is defined as chronic widespread pain and tenderness in at least 11 defined tender points for at least three months [1]. It is the second most common rheumatic disorder after osteoarthritis, affecting 4.7–5% of all women in European countries [2] and the USA [3]. In Saudi Arabia, FM was estimated in the special working population using English diagnostic tools of FM and the prevalence ranged between 27.1–68.4% in pharmacy professionals and students [4] and 6–8.2% in physicians in training [5].

Fibromyalgia’s specific etiology is not clearly understood [6]. Individuals with FM report an array of somatic and psychological symptoms, including pain, fatigue, headache, unrefreshing sleep, depression, and cognitive dysfunction [6]. These symptoms have a substantial effect on their physical functioning and may result in disabilities, leading to limited social participation [7,8]. Moreover, FM has a massive impact on an individual’s quality of life [9,10,11], productivity, and absenteeism [9,10,11], with a substantial economic burden due to healthcare [12,13,14]. Other conditions may also coexist, such as osteoarthritis, inflammatory arthritis, systemic lupus erythematosus, irritable bowel syndrome, anxiety, depression, and headache [15,16].

Patients with FM often lack a diagnosis for years due to discrepancies in recognizing the symptoms and its validity as a diagnosis [17]. Since FM has an array of symptoms with diverse rheumatologic, medical, or psychological comorbid conditions, it is a diagnostic challenge for healthcare practitioners (HCPs). Diagnosis is primarily performed clinically by HCPs using the 1990 ACR criteria [18]. The 1990 ACRs tender point examination is difficult, rarely performed, and often incorrectly conducted by HCPs [19]. FM diagnosis has evolved with the development of practical diagnostic tools (such as the London Fibromyalgia Epidemiology Study Screening Questionnaire (LFESSQ) [20], and the Fibromyalgia Survey Questionnaire (FSQ) [21]). One of those instruments is the Fibromyalgia Rapid Screening Tool (FiRST), which was developed by Perrot et al., and overcame the challenge of tender point examination and made it possible to diagnose FM using simple self-reported questionnaires in patients with diffuse chronic pain [22]. The FiRST consists of six questions related to different dimensions related to FM (widespread pain, fatigue, pain characteristics, non-painful abnormal sensations, functional somatic symptoms, sleep, and cognitive problems) and requires yes or no responses [22]. Each item corresponds to one point, and a cut-off score of 5–6 is considered positive for FM [22]. The tool can detect FM in both daily clinical practice and research [22]. The tool has been previously translated into several languages [23]. According to the MAPI Research Trust website, the FiRST instrument has not been translated into Arabic Saudi Arabia, and nor has it had its measurement properties evaluated.

Increased awareness of FM-related socioeconomic burdens has led to a rise in epidemiological studies in the general or specific population. However, epidemiological data of FM in the general population of Saudi Arabia is lacking due to the absence of translated and validated patient reported diagnostic tools. The available epidemiological data in Saudi Arabia used the original FM diagnostic tools in English language and applied it to certain working populations that were able to read and write in English [4,5]. This led to diagnosis being solely based on clinical investigations using the ACR criteria, which is challenging to HCPs. Moreover, FM is variously mis- or under-diagnosed [24]. The lack of a translated, valid, and reliable diagnostic questionnaire to be used in specific countries or cultures might led to the mis- or under-diagnosis. Therefore, this study aimed to translate the Fibromyalgia Rapid Screening Tool (FiRST) into Arabic for Saudi Arabia.

## 2. Methods

### 2.1. Ethical Considerations, Permissions, and Approvals

The process of translation and validation from the source language tool (FiRST) began after the lead author’s permission was obtained, and a formal agreement was signed. This was conducted through the Mapi Institute. Stepwise translation and validation were performed in accordance with the Mapi’s linguistic validation manual. For ethical standards, the cognitive interviews and project translation and validation protocol were subject to approval from the Institutional Review Board (approval number E-21-5885). All participants consented before inclusion.

### 2.2. Instrument Items and Data Collection Tool

The FiRST tool consisted of six yes or no questions that covered several domains [22], which included pain all over the body, “I have pain all over my body”, presence of an unpleasant general fatigue, “My pain is accompanied by a continuous and very unpleasant general fatigue”, pain sensation that resembled a burn, electric shock, or cramps, “My pain feels like burns, electric shocks or cramps”, pain associated with needles, tingling, or a numbing sensation, “My pain is accompanied by other unusual sensations throughout my body, such as pins and needles, tingling or numbness”, pain associated with other chronic problems in digestion, urination, headache or restless leg, “My pain is accompanied by other health problems such as digestive problems, urinary problems, headaches or restless legs”, and pain that affected the ability to sleep and concentrate and made the person generally slower, “My pain has a significant impact on my life, particularly on my sleep and my ability to concentrate, making me feel slower generally”. The tool was a short, self-administered questionnaire. Other data collected in a predesigned sheet included demographic data, such as age, sex, marital status, education level, years from the start of symptoms of FM, first diagnosis, family history of FM, and any previous medical history of trauma before the occurrence of FM. As associations were found in previous studies between a decreased ferritin level [25], vitamin B and D [26], with the etiology of FM, data on previous history of vitamin D or B, or ferritin deficiency were also collected.

### 2.3. Phases of Translation

The original tool was translated into Arabic by two authors (GAA and HMA) who are PhD holders, native in Arabic, and fluent in the source language (English). One was (HMA) experienced in managing patients with FM within the rheumatology clinic. The translators produced two independent translated forms: T1 (by GAA) and T2 (by HMA). After a meeting and discussion with the local coordinator (SSA), discrepancies were resolved and a pooled version (version 1) was produced. Version 1 was sent to one certified professional translator (translator 1) and then to another (translator 2) for backward translation to the source language. Both were native English speakers, bilingual in the target language, and they did not have access to the original version of the FiRST. Subsequently, the local coordinator (SSA) revised the back-translated version and compared it with the source instrument. Any differences or issues were resolved with the professional translators and version 2 was created.

**Figure 1:**
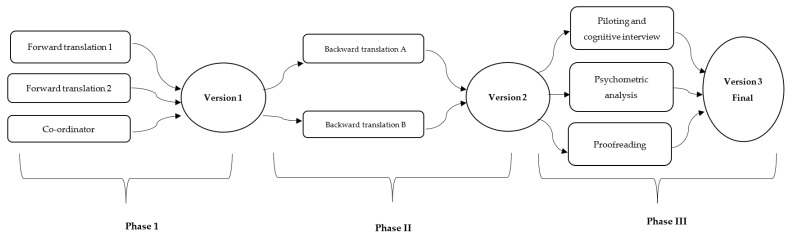
The translation and validation process of FiRST.

Version 2 was piloted and subjected to cognitive interviews with patients with FM to confirm its clarity. Finally, the last version (version 3) was proofread by a person who specialized in Arabic to check for spelling, grammar, punctuation, and typography. A methodological representation of the translation process is shown in Figure 1. In addition, any discrepancies between the forward and backward translated versions were recorded and modified upon consensus between the research team. All the results of the phases were reported to the instrument author through the Mapi Research Institute. In Phase 3, cognitive interviews, psychometric analysis, and proofreading were performed simultaneously.

### 2.4. Participant’s Recruitment

In addition to social media through the FM support groups, adult participants (aged 18 years and above) with a confirmed clinical diagnosis of FM based on the 2016 fibromyalgia diagnostic criteria were recruited from a rheumatology clinic at the Medical City in King Saud University [27]. Patients with FM aged less than 18 years old or those who were unable to read and write were excluded from the study. The recruited participants provided consent and contact details for interviews. The social media support group consisted of 110 patients with FM. An invitation letter link with contact details was sent through Twitter, and the respondents were contacted by phone and interviewed through Zoom after they consented. Patients were able to visualize the instrument and read it. Investigators were available to assist the patients if required. All participants were interviewed twice after an interval of two weeks for an assessment of within-subject reliability.

### 2.5. Sample Size, Statistical Analysis, Psychometric Properties, and Reliability

In the literature, no consensus exists to define sample size for assessing psychometric properties [28]. Moreover, no requirements were defined for sample size determination in the International Society for Quality of Life Research (ISOQOL) [29]. Therefore, sample size calculation was not considered, and samples were collected over five months period.

The collected information and survey items were coded and entered into the Statistical Package for Social Sciences (SPSS) version 26 [30]. Means and standard deviations and medians and interquartile ranges (25th and 75th percentile values) were displayed for normally and non-normally distributed data, respectively. Categorical data were presented as numbers and percentages. The translated tool’s internal consistency (reliability) was calculated, and a cutoff point of 0.7 or more was considered as good internal consistency using Cronbach’s alpha [31]. To further evaluate the consistency of the FiRST items, stepwise consistency assessment was assessed. A test–retest correlation was used to evaluate the psychometric consistency of the tool within-subjects. A *p*-value of <0.05 indicated a statistically significant Pearson’s correlation. Exploratory factor analysis was performed after it was determined whether the data were adequate for factor analysis, Kaiser–Meyer–Olkin measure of sampling adequacy was >0.5, and the produced value was significant with a *p*-value of <0.05 [32]. To add, the intraclass correlation co-efficient (ICC) of the total scores of FiRST at the two interviews was calculated using one way ANOVA [33]. In addition, the average values of the FiRST survey items were explored using a radar chart to highlight the most influential items that affected the total score.

## 3. Results

### 3.1. Demographic Characteristics

A total of 46 participants completed the cognitive and psychometric interviews (rheumatology clinic = 27 patients approached and 25 agreed, FM support group = 21). The majority were females (91%), with a mean ± SD age of 47 ± 13.1 years, and just under half were married (47.8%) (Table 1). Regarding educational level, 52.2% had no or a low educational level. The median times (IQR) since they developed and were diagnosed with FM were 9.0 (6.0–15.0) and 4.0 (2.0–7.0) years, respectively. Of these, 10 patients (21.7%) had a family history of FM. Furthermore, 59% of the participants developed FM after a trauma (58.7%). A positive history of vitamin D, B12, and ferritin deficiencies that could contribute to FM symptoms was observed in 83%, 37%, and 52.2% of the patients, respectively.

### 3.2. Translation Process Results

During the translation process, the survey items were either subject to minor or major edits. Major edits were when a word had to be changed to reflect the cultural appropriate expression, for example “ligament” was changed to “tendon” as it was more understood by the population. Another major change was that the word “other” was added, and only four statements were subject to major changes. The remainder were minor changes. Furthermore, the psychometric analysis was performed using version 2.

#### 3.2.1. Forward Translation

The forward translation was almost identical regarding the words; however, minor differences in the sentence structure were observed. Therefore, the most appropriate sentence structure was chosen after a discussion with the research team.

In the word translation, the word “tendon” in the first instruction paragraph, “You have been suffering from joint, muscle or tendon pain” [22], was translated to (arbetah) and (awtar) by the two translators. The research team discussed and chose the word (“tendon”, arbetah) and decided to revise it after the backward translation. In the second instruction paragraph, “Please fill in this questionnaire by answering either yes or no [22], the word “fill” was translated to (melae) and (taebea’a). The word (“fill”, taebea’a) was chosen. In the second item of the FiRST, the word “fatique” was translated as (wahan) and (erhaq) by the two translators. After discussion, the word (“fatique”, wahan) was chosen as the terminology was commonly used by the target population. In the third item, two words, (tashangat) and (taqalosat), were used for “cramps”. The word (“cramps”, taqalosat) was chosen after discussion. In the fifth item, one translator added the word “syndrome” to restless leg, which was not available in the original and second translators’ version. Therefore, the second translators’ version was chosen.

The research team reached a consensus on one reconciled forward translation (version 1) for the FiRST, which was ready for back translation.

#### 3.2.2. Backward Translation

The majority of the inconsistencies between the original English and back-translated versions were related to the use of synonyms and missing words in version 1.

In the first instruction paragraph, “You have been suffering from joint, muscle or tendon pain”, the translator translated it to “ligaments” rather than “tendon” as in the original tool [22]. The coordinator revised version 1 and changed the word to (“tendon”, awtar). The professional translators were contacted to translate the new modification, and this was similar to the original version “tendon”. Furthermore, the backward translation was missing the word “your”; we checked the combined version (version 1), and that was the reason for this translation. Version 1 was modified accordingly, and the translator was contacted to translate the new modification, which was similar to the original version. In the second paragraph, “Please fill in this questionnaire by answering either yes or no (only 1 answer: YES or NO) to each of the following statements. Put a cross in the box that corresponds to your answer”, the backward translation was without the word “very”. We checked version 1, and the word was missing. Version 1 was modified accordingly, and the translators were contacted to translate the new modification to be similar to the original version. For the fourth survey item, the backward version was without the word “other”. The research team checked version 1, which was the reason for this inaccuracy. Version 1 was modified accordingly, and the translator was contacted to translate the new modification, which was similar to the original version.

The research team reached a consensus on one reconciled backward translation (version 2) for the FiRST, which was ready for piloting and cognitive interviews.

### 3.3. Piloting, Cognitive Interviews, and Proofreading

Piloting (*n* = 5) followed by cognitive interviews (*n* = 46) were performed using version 2 (Figure 1). During the pilot study, three words were not clear to the participants in the translated version. The first two were “fatigue” and “numbness”, respectively. However, the research team made no changes to the translated version. The other word was “restless leg”, as reported by two participants. Hence, an extra word (khadar) was added between brackets to make it clearer and was approved by the research team. Minor grammatical changes were made during proofreading. The pilot sample was included after the cognitive interviews. No changes were recommended after cognitive interviews as participants answered the questionnaire smoothly. Therefore, the survey results were subjected to a psychometric analysis.

The final version (version 3) can be obtained with permission from the MAPI Research Trust, Lyon, France. E-mail: PRO information@mapi-trust.org; http://www.mapi-trust.org (accessed on 26 April 2021).

### 3.4. Psychometric Properties

**Figure 2:**
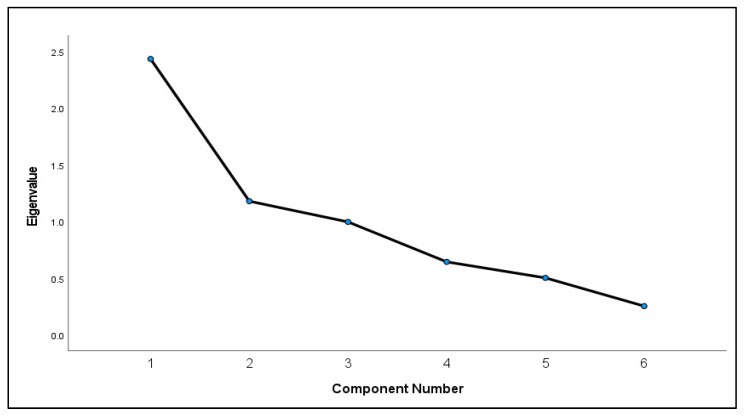
Factor analysis of the FiRST survey items with Eigenvalue (N = 46).

**Figure 3:**
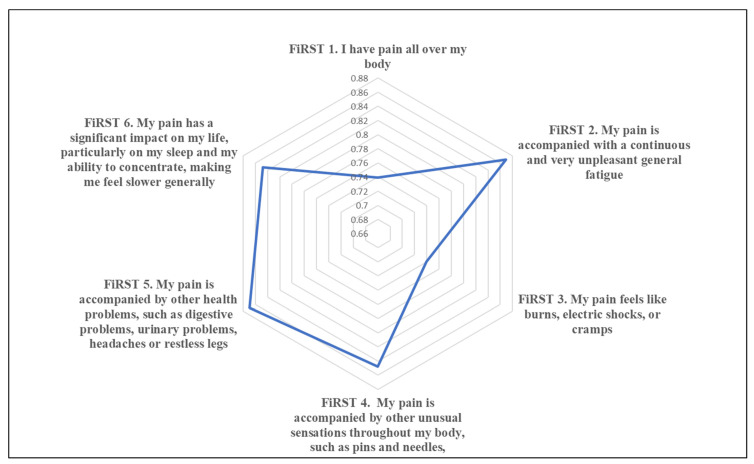
Radar chart for FiRST survey items of the 46 participants.

This step was performed using version 2. The survey had good internal consistency, with a Cronbach’s alpha of 0.7. Stepwise consistency assessment results were as follows: after removing item or question six (Q6), Q5, Q4, Q3, Q2, and Q1 of FiRST, the Cronbach’s alpha was 0.6, 0.7, 0.7, 0.6, 0.6, and 0.6, respectively. In addition, 78% of the sample completed the retest after two weeks. A significant test–retest correlation was found, with a Pearson’s correlation coefficient and a *p*-value of 0.79 and 0.001, respectively. In addition, the ICC of the FiRST total score is 0.834, indicating high test–retest reliability. The data were adequate to perform a factor analysis, with a Kaiser–Meyer–Olkin measure of sampling adequacy of 0.651 and a *p*-value of 0.001 (Table 2). Two factors, namely, items or components one (general symptoms) and two (specific sensations), were identified with an eigenvalue of > 1 (Figure 2). In the radar chart, the least expected values to be answered with “yes” were items one and three in the FiRST (Figure 3), which indicated that participants were likely to answer “no” for these two items.

## 4. Discussion

This study was the first to translate and validate the FiRST diagnostic questionnaire for FM in the Arabic language. A forward–backward translation process was performed. Internal consistency, reliability, and factor analysis were performed to assess the psychometric properties of 46 patients with FM. The results of the cognitive interviews indicated that the Arabic translations of the FiRST accurately captured the concepts of the English version. These findings suggest that the translated Arabic version has good internal consistency and reliability and is suitable for screening in practice.

The literature found that the time it takes to establish a definitive diagnosis of FM usually takes several years, leading to numerous consultations and investigations with a high personal and societal burden [7,34,35]. In our study, patients took a median of four years to receive a definitive diagnosis of FM. Awareness and knowledge of HCPs are key to accelerating the diagnosis of patients. Several previous studies have examined the knowledge of HCPs, including rheumatologists, general practitioners, and medical students, and found a large variation in the knowledge of diagnosing FM in different specialties [36,37,38,39,40,41]. In the Saudi context, Kaki et al. and Alodaibi et al. investigated the awareness of medical practitioners and physical therapist and their findings were consistent with other studies’ findings in terms of the lack of knowledge and awareness of FM [42,43].

The availability of an accessible, translated, and validated diagnostic questionnaire can be considered another key factor to accelerate the diagnosis of patients with FM and prevent patients from suffering for a long time with their pain and symptoms [44]. As mentioned in the introduction, the lack of translated and validated FM diagnostic tools to be used in different countries and cultures has led to diagnoses being solely based on clinical investigations using the ACR criteria, which is challenging for HCPs. In this perspective, the FiRST is one of the instruments that is short, simple, and easy to be conducted by HCPs. Furthermore, it can overcome the challenge of the tender point examination being performed using another diagnostic tool, such as the 1990 ACR criteria [18,22]. Apart from the French and the English versions of FiRST [22], FiRST has been translated according to MAPI Research Trust website into Dutch, Belgium, German, Greek, Italian, Portuguese, Russian, Spanish, Turkish, and Ukrainian languages [23]. The linguistic validation was not applied to all translations [23], and limited to the Spanish [45], Greek [46], Brazilian [47], and Turkish [48] versions. The Arabic translation and validation of the FiRST have not been investigated previously.

We compared our findings with other validated Spanish [45], Greek [46], Brazilian [47], and Turkish [48] versions of FiRST. In the Spanish study conducted by Torres and colleagues [45], participants with suspected FM (*n* = 172) were included in the study and were compared to a group of controls (non-FM = 85). They performed a stepwise translation process similar to ours. The Cronbach’s alpha for the internal consistency of the Spanish version was 0.72, which was similar to our Arabic version. The Spanish translation included other surveys to detect confounders that might affect the validity of the Spanish FiRST version. For the global score of Spanish FiRST, the ICC was very strong (0.76, *p*-value < 0.01), which was also similar to our finding. In the Zis et al. study that translated and validated FiRST into the Greek language [46], they included fewer FM patients than our sample (*n* = 42), and they compared them to a control group of patients with osteoarthritis (*n* = 59). The Cronbach’s alpha for the internal consistency was 0.79, which is similar to what we reported in the Arabic version; the ICC was also high (0.955). The Brazilian study translated and validated an online version of FiRST on two groups of patients; one group had chronic pain and the other group had confirmed diagnosis of FM [47]. In addition to the FiRST, multiple surveys assessing pain and fatigue were used. In the first step to assess the translated Brazilian version, the questionnaire was sent to healthcare professionals (*n* = 10) and female patients with FM (*n* = 20). The agreement between the ACR 2016 diagnostic criteria and the FiRST was high, indicating the reliability of the FiRST. The study was cross-sectional and had the benefit of comparison with a control group. However, the internal consistency was not assessed, and it was calculated by another Brazilian study with a Cronbach’s alpha of > 0.73 [49]. The Turkish version of the FiRST [48] was subject to translation and validation with a very similar methodology to the one used by us. The authors conducted three phases of the translation process. The total score of the survey was then linked to other scores, one of which was the Hospital Anxiety and Depression Scale (HADS). A high correlation between the HADs and the FiRST was found, indicating a good reliability of the translated FiRST. A high test–retest reliability coefficient (0.875) was also found. Although the study has a strong, constructed methodology and recruited a large number of patients (*n* = 269), the time between applications of the FiRST to patients for test–retest was only six hours.

The major strength of the current study was the translation process, which followed the MAPI linguistic validation manual, that recommended an independent translator who was a native English speaker. In our study, we included two native English speakers for the backward translation, which was supported by another translation guideline [50]. Moreover, to ensure transparency, every step was detailed, and each change was documented and reported to MAPI.

A limitation of the current study was the small number of participants, which reflected the small number of patients diagnosed with FM. This was consistent with a study in Saudi Arabia, which found that more than half of the physical therapists mentioned that the number of patients treated for FM was less than five per year [43]. In addition, difficulties in recruiting patients affected the sample size. The severity of their pain might impact their willingness to participate [51]. The recruitment of patients through two approaches (rheumatology clinic and FM support group) may be considered as a strength and could introduce diversity in the sample population, as one might expect that clinical patients might have more severe cases. Moreover, participants from the clinic were older (mean age: 52 years vs. 43 years) and had more females compared to the FM support group (95% vs. 87%).

Further analysis with a larger sample size of Saudi patients at different stages of the disease is recommended. Further research is also required to confirm the translation adequacy and psychometric properties in other Arabic-speaking populations. Psychometric analyses that were not conducted in our study, such as criterion and construct validity and responsiveness, also require further research. In conclusion, the Arabic FiRST exhibited good internal consistency, reliability, and validity for detecting patients with FM in Saudi Arabia. Furthermore, its use in clinical practice is recommended.

## Figures and Tables

**Table 1:** Participants’ demographic characteristics.

Demographic Characteristic		N = 46
Gender, n (%)	Male	4 (8.7)
	Female	42 (91.3)
Age in years, mean (SD)		47.2 (13.1)
Marital, n (%)	Married	22 (47.8)
Education, n (%)	No to low education (primary or secondary school or high school)	24 (52.2)
	Higher education (university or diploma or post graduate studies)	21 (46.7)
Years from the first diagnosis of fibromyalgia, median (IQR)		4.0 (2.0–7.0)
Years from the start of symptoms of fibromyalgia, median (IQR)		9.0 (6.0–15.0)
Positive family history of fibromyalgia or any rheumatic disorder, n (%)		10 (21.7)
Positive history of trauma before the occurrence of fibromyalgia, n (%)		27 (58.7)
Positive history of vitamin D deficiency, n (%)		38 (82.6)
Positive history of vitamin B 12 deficiency, n (%)		17 (37.0)
Positive history of iron or ferritin deficiency, n (%)		24 (52.2)

Note: IQR: interquartile range. SD: standard deviation.

**Table 2:** Psychometric properties of the survey items.

	Value or Coefficient	*p*-Value if Available
Participants with test, number (%)	46 (100)	
Mean (standard deviation) value of test FiRST items	4.9 (1.4)	
Participants with re-test, number (%)	36 (78.3)	
Mean (standard deviation) of re-test FiRST items	5.1 (1.4)	
Test–re-test coefficient	0.793	< 0.001 *
Test for internal consistency Cronbach’s Alpha	0.7	
Intera-class correlation co-efficient	0.834	< 0.001 *
Kaiser–Meyer–Olkin Measure of Sampling Adequacy	0.651	< 0.001 *

Note: * Significant value was *p* < 0.050.

## Data Availability

Data available on request.

## References

[1] Wolfe F., Smythe H.A., Yunus M.B., Bennett R.M., Bombardier C., Goldenberg D.L., Tugwell P., Campbell S.M., Abeles M., Clark P. (1990). The American College of Rheumatology 1990 Criteria for the Classification of Fibromyalgia. Arthritis Rheum..

[2] Branco J.C., Bannwarth B., Failde I., Abello Carbonell J., Blotman F., Spaeth M., Saraiva F., Nacci F., Thomas E., Caubere J.-P. (2010). Prevalence of Fibromyalgia: A Survey in Five European Countries. Semin. Arthritis Rheum..

[3] Lawrence R.C., Felson D.T., Helmick C.G., Arnold L.M., Choi H., Deyo R.A., Gabriel S., Hirsch R., Hochberg M.C., Hunder G.G. (2008). Estimates of the Prevalence of Arthritis and Other Rheumatic Conditions in the United States. Part II. Arthritis Rheum..

[4] AlAujan S.S., Almalag H.M., Omair M.A. (2021). Prevalence of Fibromyalgia in Pharmacy Professionals and Students: A Cross-Sectional Study. J. Pain Res..

[5] Omair M.A., Alobud S., Al-Bogami M.H., Dabbagh R., Altaymani Y.K., Alsultan N., Alhazzani A., Omair M.A. (2019). Prevalence of Fibromyalgia in Physicians in Training: A Cross-Sectional Study. Clin. Rheumatol..

[6] Rahman A., Underwood M., Carnes D. (2014). Fibromyalgia. BMJ.

[7] Choy E., Perrot S., Leon T., Kaplan J., Petersel D., Ginovker A., Kramer E. (2010). A Patient Survey of the Impact of Fibromyalgia and the Journey to Diagnosis. BMC Health Serv. Res..

[8] Ullrich A., Farin E., Jackel W.H. (2012). Restrictions in participation in women with fibromyalgia syndrome. An explorative pilot study. Schmerz.

[9] Grodman I., Buskila D., Arnson Y., Altaman A., Amital D., Amital H. (2011). Understanding Fibromyalgia and Its Resultant Disability. Isr. Med. Assoc. J..

[10] Mannerkorpi K., Gard G. (2012). Hinders for Continued Work among Persons with Fibromyalgia. BMC Musculoskelet. Disord..

[11] White L.A., Birnbaum H.G., Kaltenboeck A., Tang J., Mallett D., Robinson R.L. (2008). Employees with Fibromyalgia: Medical Comorbidity, Healthcare Costs, and Work Loss. J. Occup. Environ. Med..

[12] Wolfe F., Anderson J., Harkness D., Bennett R.M., Caro X.J., Goldenberg D.L., Russell I.J., Yunus M.B. (1997). A Prospective, Longitudinal, Multicenter Study of Service Utilization and Costs in Fibromyalgia. Arthritis Rheum..

[13] Robinson R.L., Birnbaum H.G., Morley M.A., Sisitsky T., Greenberg P.E., Claxton A.J. (2003). Economic Cost and Epidemiological Characteristics of Patients with Fibromyalgia Claims. J. Rheumatol..

[14] Hughes G., Martinez C., Myon E., Taïeb C., Wessely S. (2006). The Impact of a Diagnosis of Fibromyalgia on Health Care Resource Use by Primary Care Patients in the UK: An Observational Study Based on Clinical Practice. Arthritis Rheum..

[15] Weir P.T., Harlan G.A., Nkoy F.L., Jones S.S., Hegmann K.T., Gren L.H., Lyon J.L. (2006). The Incidence of Fibromyalgia and Its Associated Comorbidities: A Population-Based Retrospective Cohort Study Based on International Classification of Diseases, 9th Revision Codes. J. Clin. Rheumatol..

[16] Cohen H. (2017). Controversies and Challenges in Fibromyalgia: A Review and a Proposal. Ther. Adv. Musculoskelet. Dis..

[17] Hayes S.M., Myhal G.C., Thornton J.F., Camerlain M., Jamison C., Cytryn K.N., Murray S. (2010). Fibromyalgia and the Therapeutic Relationship: Where Uncertainty Meets Attitude. Pain Res. Manag..

[18] Glennon P. (2010). Fibromyalgia Syndrome: Management in Primary Care. Rep. Rheum. Dis..

[19] Fitzcharles M., Boulos P. (2003). Inaccuracy in the Diagnosis of Fibromyalgia Syndrome: Analysis of Referrals. Rheumatology.

[20] White K.P., Harth M., Speechley M., Ostbye T. (1999). Testing an Instrument to Screen for Fibromyalgia Syndrome in General Population Studies: The London Fibromyalgia Epidemiology Study Screening Questionnaire. J. Rheumatol..

[21] Häuser W., Jung E., Erbslöh-Möller B., Gesmann M., Kühn-Becker H., Petermann F., Langhorst J., Weiss T., Winkelmann A., Wolfe F. (2012). Validation of the Fibromyalgia Survey Questionnaire within a Cross-Sectional Survey. PLoS ONE.

[22] Perrot S., Bouhassira D., Fermanian J. (2010). Development and Validation of the Fibromyalgia Rapid Screening Tool (FiRST). Pain.

[23] Mapi Research Trust Fibromyalgia Rapid Screening Tool (FiRST). https://eprovide.mapi-trust.org/instruments/fibromyalgia-rapid-screening-tool.

[24] Häuser W., Sarzi-Puttini P., Fitzcharles M.-A. (2019). Fibromyalgia Syndrome: Under-, over- and Misdiagnosis. Clin. Exp. Rheumatol..

[25] Ortancil O., Sanli A., Eryuksel R., Basaran A., Ankarali H. (2010). Association between Serum Ferritin Level and Fibromyalgia Syndrome. Eur. J. Clin. Nutr..

[26] Munipalli B., Strothers S., Rivera F., Malavet P., Mitri G., Abu Dabrh A.M., Dawson N.L. (2022). Association of Vitamin B12, Vitamin D, and Thyroid-Stimulating Hormone With Fatigue and Neurologic Symptoms in Patients with Fibromyalgia. Mayo Clin. Proc. Innov. Qual. Outcomes.

[27] Wolfe F., Clauw D.J., Fitzcharles M.-A., Goldenberg D.L., Hauser W., Katz R.L., Mease P.J., Russell A.S., Russell I.J., Walitt B. (2016). 2016 Revisions to the 2010/2011 Fibromyalgia Diagnostic Criteria. Semin. Arthritis. Rheum..

[28] Anthoine E., Moret L., Regnault A., Sébille V., Hardouin J.-B. (2014). Sample Size Used to Validate a Scale: A Review of Publications on Newly-Developed Patient Reported Outcomes Measures. Health Qual. Life Outcomes.

[29] Reeve B.B., Wyrwich K.W., Wu A.W., Velikova G., Terwee C.B., Snyder C.F., Schwartz C., Revicki D.A., Moinpour C.M., McLeod L.D. (2013). ISOQOL Recommends Minimum Standards for Patient-Reported Outcome Measures Used in Patient-Centered Outcomes and Comparative Effectiveness Research. Qual. Life Res..

[30] IBM Corp (2021). IBM SPSS Statistics for Windows.

[31] Tavakol M., Dennick R. (2011). Making Sense of Cronbach’s Alpha. Int. J. Med. Educ..

[32] Kaiser H.F. (1974). An Index of Factorial Simplicity. Psychometrika.

[33] Shrout P.E., Fleiss J.L. (1979). Intraclass Correlations: Uses in Assessing Rater Reliability. Psychol. Bull..

[34] Briones-Vozmediano E., Vives-Cases C., Ronda-Pérez E., Gil-González D. (2013). Patients’ and Professionals’ Views on Managing Fibromyalgia. Pain Res. Manag..

[35] Häuser W., Zimmer C., Felde E., Köllner V. (2008). What Are the Key Symptoms of Fibromyalgia? Results of a Survey of the German Fibromyalgia Association. Der. Schmerz..

[36] Arshad A., Ooi K.K. (2007). Awareness and Perceptions of Fibromyalgia Syndrome: A Survey of Southeast Asian Rheumatologists. J. Clin. Rheumatol..

[37] Blotman F., Thomas E., Myon E., Andre E., Caubere J.P., Taieb C. (2005). Awareness and Knowledge of Fibromyalgia among French Rheumatologists and General Practitioners. Clin. Exp. Rheumatol..

[38] Mu R., Li C., Zhu J., Zhang X., Duan T., Feng M., Wang G., Zhang F., Li Z. (2013). National Survey of Knowledge, Attitude and Practice of Fibromyalgia among Rheumatologists in China. Int. J. Rheum. Dis..

[39] Kumbhare D., Ahmed S., Sander T., Grosman-Rimon L., Srbely J. (2018). A Survey of Physicians’ Knowledge and Adherence to the Diagnostic Criteria for Fibromyalgia. Pain Med..

[40] Acuña Ortiz F.E., Capitán de la Cruz V.A., León Jiménez F.E. (2017). Knowledge on Fibromyalgia Among General Practitioners, From Chiclayo-Peru, 2016. Reumatol. Clin..

[41] Amber K.T., Brooks L., Chee J., Ference T.S. (2014). Assessing the Perceptions of Fibromyalgia Syndrome in United States Among Academic Physicians and Medical Students: Where Are We and Where Are We Headed?. J. Musculoskelet. Pain.

[42] Kaki A.M., Hazazi A.A. (2018). Assessment of Medical Practitioners’ Knowledge of Fibromyalgia in Saudi Arabia. Saudi J. Anaesth..

[43] Alodiabi F., Alhowimel A., Alotaibi M., Alamam D., Fritz J.M. (2020). Knowledge, Awareness, and Perceptions of the Diagnosis and Management of Fibromyalgia Among Physical Therapists in Saudi Arabia: A Cross-Sectional Survey. Open Access Rheumatol..

[44] Wolfe F. (2009). Fibromyalgianess. Arthritis Rheum..

[45] Torres X., Collado A., Gomez E., Arias A., Cabrera-Villalba S., Messina O.D., Vidal L.F., Clark P., Ríos C., Salomon P.A. (2013). The Spanish Version of the Fibromyalgia Rapid Screening Tool: Translation, Validity and Reliability. Rheumatology.

[46] Zis P., Brozou V., Stavropoulou E., Argyra E., Siafaka I., Kararizou E., Bouhassira D., Perrot S., Zis V., Vadalouca A. (2017). Validation of the Greek Version of the Fibromyalgia Rapid Screening Tool. Pain Pract..

[47] Sousa A., Arruda G., Pontes-Silva A., Souza M.C., Driusso P., Avila M. (2022). Measurement Properties of the Brazilian Online Version of the Fibromyalgia Rapid Screening Tool (FiRST). Adv. Rheumatol..

[48] Celiker R., Altan L., Rezvani A., Aktas I., Tastekin N., Dursun E., Dursun N., Sarıkaya S., Ozdolap S., Akgun K. (2017). Reliability and Validity of the Turkish Version of the Fibromyalgia Rapid Screening Tool (FiRST). J. Phys. Ther. Sci..

[49] Daltrozo J.B., Paupitz J.A., Neves F.S. (2020). Validity of Fibromyalgia Survey Questionnaire (2016) Assessed by Telephone Interview and Cross-Cultural Adaptation to Brazilian Portuguese Language. Adv. Rheumatol..

[50] Beaton D., Bombardier C., Guillemin F., Ferraz M.B. (2007). Recommendations for the Cross-Cultural Adaptation of the DASH & QuickDASH Outcome Measures.

[51] Browne J.L., Rees C.O., van Delden J.J.M., Agyepong I., Grobbee D.E., Edwin A., Klipstein-Grobusch K., van der Graaf R. (2019). The Willingness to Participate in Biomedical Research Involving Human Beings in Low-and Middle-income Countries: A Systematic Review. Trop. Med. Int. Health.

